# Prevalence, incidence and associated factors of musculoskeletal disorders before and during the Covid-19 pandemic in faculty members: a comparative cross-sectional study

**DOI:** 10.1186/s12891-024-07820-4

**Published:** 2024-09-02

**Authors:** Mahdi Jalali, Sajjad Farhadi, Reza Esmaeili, Hamed Aghaei, Somayeh Rahimimoghadam, Mahdieh Niroumand, Aida Shahmohammadi

**Affiliations:** 1 Department of Occupational Health and Safety Engineering, Workplace Health Research Center, Faculty of Health, Neyshabur University of Medical Sciences, Neyshabur, Iran; 2 Department of Occupational Health and Safety Engineering, School of Health, Isfahan University of Medical Sciences, Isfahan, Iran; 3 Department of Occupational Health and Safety Engineering, School of Public Health, Islamic Azad Tehran Medical Sciences University, Tehran, Iran; 4 Department of Occupational Health Engineering, School of Health, Neyshabur University of Medical Sciences, Neyshabur, Iran

**Keywords:** Musculoskeletal disorders, Distance education, COVID-19, Ergonomics, Prevalence study, Incidence

## Abstract

**Background:**

The COVID-19 pandemic has led to the broad acceptance of distance education (DE), with university professors and students conducting the teaching–learning process remotely from their homes. The propose of this study to investigate the prevalence of musculoskeletal disorders (MSDs) before and during the COVID-19 pandemic and identify risk factors associated with DE that may contribute to an increased incidence of these disorders among university professors.

**Methods:**

This cross-sectional analytical study took a comparative approach and involved 310 university professors in Iran. Data were gathered using an online questionnaire. Initially, demographic and occupational information of the professors, hours of physical activity, and hours spent using electronic devices were recorded. Participants were then asked to report MSDs in various body areas throughout the previous year and the previous seven days. Finally, MSDs risk factors such as workstation ergonomics during computer, laptop, smartphone, and tablet use, as well as working postures during online teaching or offline content development during the COVID-19 pandemic, were examined.

**Results:**

The majority of the participants were male (66.13%), with a PhD (46.77%) and a faculty member position (74.2%). On average, the use of computers and laptops increased by 2.67 h and 2.72 h, respectively, during the pandemic compared to before the pandemic. This increase was statistically significant (*P* < 0.001). MSDs incidence increased significantly before and during the COVID pandemic was observed in the areas of the neck, shoulders, lower and upper back, arms, forearms, wrists and fingers (*P* < 0.05). The highest cumulative incidence (Cin) of MSDs was related to the neck (Cin = 24.20%), upper back (Cin = 21.29%), low back (Cin = 18.06%) and fingers (Cin = 16.13%). The prevalence of MSDs during the COVID pandemic was significantly associated with employment status (*P* = 0.042), work experience (*P* = 0.016), age (*P* = 0.027), increase in the use of computers/ laptops (*P* < 0.001), decrease of the smartphone/tablet distance from the body (*P* = 0.047), workstation (smartphone-tablet, computer, laptop) (*P* < 0.05), head position (smartphone-tablet) (*P* = 0.029), display height (computer/laptop) (*P* = 0.045) and physical activity (*P* = 0.006).

**Conclusions:**

It appears that the increased duration of smartphone, computer, and laptop use, combined with decreased physical activity and detrimental changes in ergonomic conditions of workstations during the quarantine period caused by the COVID-19 pandemic, has resulted in a shift from dynamic to static tasks and an increase in the prevalence and incidence of MSDs among university professors.

## Introduction

In late December 2019, the first cases of individuals exhibiting respiratory symptoms were diagnosed in Wuhan, China. The viral illness, caused by an unidentified microbial agent, was named Novel Coronavirus 2019 (SARS-CoV-2/COVID-19). It rapidly became a pandemic, spreading worldwide [1, 2]. In March 2020, the World Health Organization (WHO) declared COVID-19 as a global pandemic. WHO and public health authorities worldwide took measures to control the spread of COVID-19 [3]. In Iran, the first confirmed case of COVID-19 was reported on February 16, 2020, in the city of Qom. The rapid escalation of this acute respiratory syndrome led to quarantine measures in many affected countries. By February 20, 2020, Iran's status changed from green to yellow, and by March 3, 2020, the disease had rapidly spread throughout the country [4]. According to the Iranian Ministry of Health's report, as of noon on March 3, 2020, the total number of confirmed COVID-19 cases reached 2,922, with 92 fatalities. Restrictions were imposed nationwide on February 20, 2020. These gradual restrictions included the closure of kindergartens, schools, universities, restaurants, shopping centers, social and economic activity centers, and beauty salons. These closures continued in several phases in Iran until the end of the Iranian calendar year 1400 (March 20, 2021) [5].

With the coronavirus outbreak and the WHO declaration of the COVID-19 pandemic, many institutions, including schools and universities, were closed around the world to prevent the spread of infection. The global expansion of the coronavirus necessitated the implementation of quarantine measures for almost three billion individuals as an essential approach for reducing virus transmission in most countries [6]. The onset of this disease, along with the imposition of special and unusual conditions, led to a decline in physical activity. As a result, at this time, the internet and social media were unavoidably used for a variety of professional, instructional, and educational reasons [7, 8]. Among the affected professions, schools and universities, particularly university professors, witnessed changes in their educational responsibilities as a result of actions made in several nations to deal with the COVID problem. Educational activities were carried out remotely, referred to as DE. In essence, these changes in students and teachers resulted in increased usage of social media and a shift toward virtual learning [9].

DE refers to education in a learning environment where instructors and students are separated by location, time, or both [10]. DE needs a variety of equipment, the most prevalent and commonly utilized of which are PCs, laptops, and mobile devices for virtual teaching and learning [11, 12]. With the expansion of DE during the COVID pandemic, students spent numerous hours daily attending online classes, and professors may have spent hours preparing content using these tools [13, 14]. Consequently, it seems that, following this pandemic and the created limitations, not only in the infected individuals and their close contacts but also in the entire society, the unpleasant experiences and psychological pressures associated with quarantine and DE could be examined as consequences [15]. One of the most important potential consequences of DE is the increased prevalence of MSDs, which has recently been reported in university students and faculty members [16, 17].

The extensive use of computers and social media platforms can result in changes in people's routines. When working with social media, these conditions can cause reduced mobility, increased repetitive movements, particularly in the hands and upper limbs, increased undesirable physical conditions, and prolonged static postures. This combination of characteristics is acknowledged as the most significant risk factor for the development of MSDs [18, 19]. MSDs are defined as abnormalities affecting the muscles, tendons, synovial sheaths, synovial membranes, nerves, joints, bones, ligaments, and blood vessels. They can be induced by chronic stress or an acute or unexpected trauma, resulting in pain in numerous parts of the body [20]. The upper body's neck, shoulders, back, particularly the spinal column, and upper limbs, including hands, wrists, fingers, forearms, and arms, are the most vulnerable to MSDs while using computers and social media [21]. Repetitive activities, excessive force, improper posture, contact pressures, vibrations, and physical exhaustion are among the most significant physical risk factors for MSDs in many professions [22]. In computer users, in addition to some of the mentioned factors, factors related to the design of the workstation, such as the duration of computer or social media use, individuals' rest periods, keyboard usage method, computer monitor position, type and use of devices connected to the computer, and the chair's condition also play a role in the development of MSDs [21, 23, 24].

Holidays and quarantine measures are effective ways to prevent the spread of the COVID-19 virus [25]. However, adverse effects on the musculoskeletal system may arise [16]. The increasing number of MSDs in professors not only lowers the quality of teaching and learning, but it also imposes a large economic cost on society by causing physical problems and resulting in referrals to orthopaedic specialists [26]. While many cross-sectional and epidemiological studies have examined the prevalence of MSDs in university professors during the DE period [27–34], few studies have focused on the incidence of these disorders and compared their prevalence before and during the COVID pandemic, as well as their association with potential factors that may affect professors during this period [35, 36].

The main objective of this study is to determine the prevalence of MSDs among university professors during the COVID pandemic and compare that rate to prior the COVID pandemic. Furthermore, this study attempts to identify and assess the risk factors for these disorders during the pandemic period. In this study, we hypothesized that the cumulative incidence of MSDs among university professors during the COVID pandemic and subsequent quarantines would be higher than it was prior to the pandemic. The second hypothesis was that some of the new conditions established by quarantine and DE may help reduce the occurrence of these disorders.

## Methods

### Study design

This cross-sectional analytical study took a comparison approach, employing an online survey given to professors at Iranian universities to examine data obtained before and during the COVID-19 pandemic.

### Setting

Professors at medical universities were invited to take part in this study, and an electronic questionnaire was sent to them through the Persian-language "Porsline" platform. Respondents submitted their responses via the internet.

### Ethical approval

This study was approved as a research project by the research ethics committees of Neyshabur University of Medical Sciences under the ethical code IR.NUMS.REC.1400.053 and adhered to the principles mentioned in the 'Declaration of Helsinki' (1964). All participants filled up and signed the online informed consent forms. The individuals gave their agreement to participate by answering the questionnaire. The researchers only had access to unidentified data.

### Participants

Professors from Iranian medical universities participated in this survey. Teaching at least 4 credit hours in-person every term previous to the COVID-19 pandemic, teaching at least 2 terms DE in medical universities following the pandemic's onset, and not having non-occupational MSDs (from trauma, sports injuries, accidents, and other incidents) were the criteria for enrolment. Getting COVID-19 while gathering data and terminating the study's collaboration with the medical university were among the exclusion criteria. Recently, in several studies, the prevalence of neck pain during the COVID-19 pandemic in university professors has been reported to be 50% [35, 37, 38]. The sample size was determined with a 95% confidence level and a margin of error of 0.05 using Eq. 1.

1 $$n = \frac{{(Z - {\raise0.7ex\hbox{$\alpha $} \!\mathord{\left/ {\vphantom {\alpha 2}}\right.\kern-0pt} \!\lower0.7ex\hbox{$2$}} \times P(1 - P)}}{{d^{2} }} = \frac{(1.96 \times 0.5(1 - 0.5)}{{(0.05)^{2} }} = 196$$

Given a potential 10% sample dropout, the sample size was adjusted using Eq. 2:

2 $$n_{corrected} = \frac{1}{1 - 0.1} \times 196 = 216$$

As a result, the minimum sample size was determined to be 216 people. According to the study inclusion and exclusion criteria, 310 people were examined in this study.

### Data collection

An online questionnaire was used to gather data. Three sections made up the questionnaire. 1) Details about occupation and demographics. 2) Determining the prevalence of MSDs. 3) Determining the risk factors for MSDs.

The questionnaires were created in virtual space utilizing the "Porsline" platform, which is available in Persian. Participants were sent the questionnaires through a variety of virtual groups connected to universities and national organizations. The URLs to the questionnaires were shared in groups and on people's profiles on messaging services like WhatsApp, Telegram, Eitaa, and Balleh. The participants were asked to continue filling out the questionnaires. The data was collected from the beginning of October to the end of December 2020. During this period, due to quarantine regulations in Iran, all university professors conducted DE from home. The details for each of the questionnaire’s sections are provided below.

1. *Demographic and Occupational Information*: In this section, information was acquired about the age, height, weight, gender, job experience, employment status, and educational background of participants. It also gathered data on average number of units taught, average number of hours taught, average time spent doing physical activities in minutes, and average amount of time spent using electronic devices (such as computers, laptops, tablets, and smartphones) before and during the COVID-19 pandemic.
2. *Assessment of the Prevalence of MSDs*: Participants were asked to disclose MSDs in different body parts from the previous year (before to COVID-19) and the last seven days (during the COVID-19 pandemic) in the second section. This section's data came from the Nordic questionnaire. Questions were asked about MSDs in several body parts, such as the neck, shoulders, arms, forearms, wrists, upper and lower back, buttocks, thighs, knees, and legs. The questions for each body part were to be answered with a "yes" or "no" by the participants [39]. Equation 3 was used to calculate the point prevalence of MSDs in each body location prior to and during the COVID-19 pandemic based on the response rates for each period. Point prevalence is defined as the number of observed outcome cases (individuals with a 'yes' response for each body area) at a specific point in time, divided by the total population susceptible to the outcome (population at risk).

3 $$P=\frac{C}{N}$$

Where, P represents the prevalence of MSDs at a specific point in time, C is the number of observed cases at time 't', and N is the population size at time 't'.

Equation 4 was in fact used to compute cumulative incidence. The number of new incidences of an outcome (or event) over a certain time period divided by the total population at risk at the start of that period is recognized as cumulative incidence.

4 $${C}_{i}=\frac{Nc}{Tp}$$

Where, Ci represents the cumulative incidence, Nc is the number of new cases of MSDs one year after the onset of the COVID-19 pandemic, and Tp is the population at risk at the beginning of the period.

3. *Assessment of Risk Factors for MSDs*: In the third section of the questionnaire, risk factors contributing to MSDs were addressed. These risk factors included:
  1. Brightness of the virtual learning environment.
  2. Distance from the laptop/computer.
  3. Distance from the smartphone/tablet.
  4. Workstation status when using a smartphone/tablet.
  5. Workstation status when working with a computer.
  6. Workstation status when working with a laptop.
  7. Neck position when working with a smartphone/tablet.
  8. Display height when working with a computer/laptop.
  9. Maintenance and typing methods when using a smartphone. 

Except for the variable "brightness of the workstation," all options for each variable were illustrated using guide images to help respondents make their selections (Table 1). Respondents were asked to choose their predominant working conditions during DE, which includes online teaching or preparing offline content during the COVID-19 pandemic. Additionally, participants were requested to express their overall opinion about the ergonomic conditions of the university workstation compared to the home workstation. Response options included:

1. University workstation ergonomic conditions are better.
2. Home workstation ergonomic conditions are better.
3. There is no difference in ergonomic conditions between the university and home workstations.

**Table 1:**
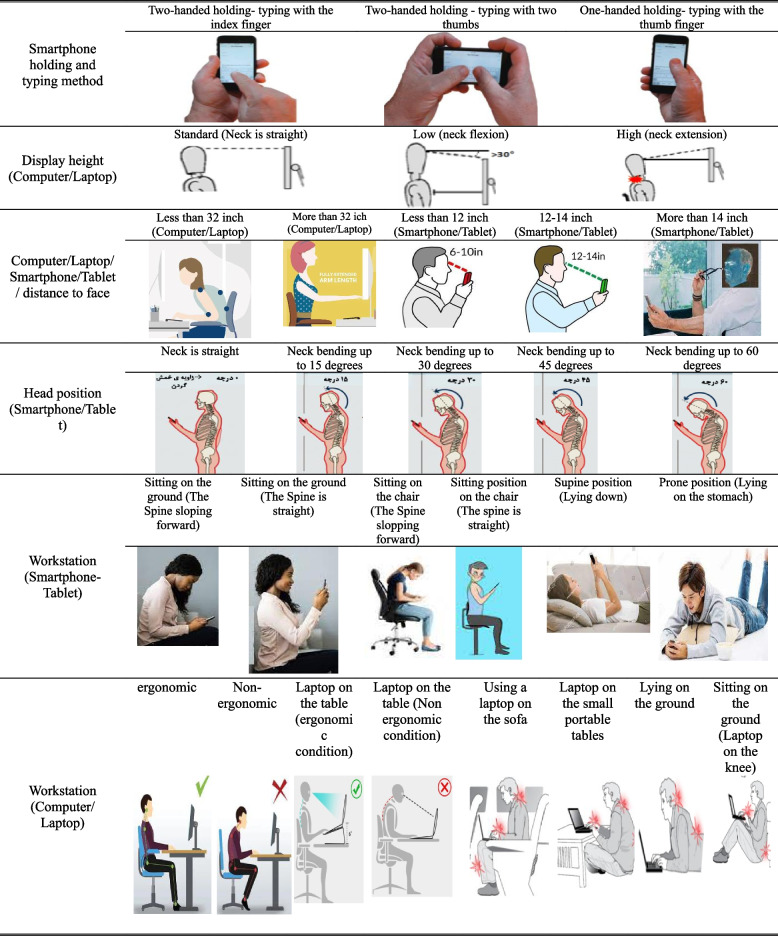
Ergonomic risk factors causing MSDs recorded in this study and guide images

### Data analysis

The statistical analysis was conducted using SPSS version 24. Descriptive statistics like mean, standard deviation, minimum, and maximum were used to describe the data. Excel 2016 and Microsoft Word were used to construct the tables and graphics. The paired sample t-test was used to compare the means of quantitative factors prior to and during the COVID-19 pandemic. The McNemar Test was used to compare the prevalence of MSDs before and during the pandemic. The chi-square test (χ2) was used to investigate the correlation between MSDs and ergonomic, demographic, and occupational risk variables. For every test, a significance level of α = 0.05 was taken into account.

## Results

Table 2 summarizes the descriptive results that detail the participants' demographic and occupational characteristics. A response rate of 53.91% was obtained from the 310 completed questionnaires that were returned to the researchers out of the 575 that were shared online. 66.13% of the participants were male, 46.77% had PhD, and 74.2% were faculty members. Age and BMI were found to have mean ± standard deviations (SD) of 42.41 ± 9.24 and 21.67 ± 4.70, respectively. Before and during COVID, the mean ± SD of teaching hours were 135.17 ± 35.89 and 128.97 ± 32.38, respectively. Notably, the number of teaching hours before and during COVID-19 did not differ statistically significantly (*P* Value = 0.636).

**Table 2:** Demographic and occupational characteristics of the participants (*n* = 310)

**Variable**	Mean ± SD	**Range**	
Age (Year)	42.41 ± 9.24	32–60	
Height (cm)	167.95 ± 8.27	146–190	
Weight (kg)	69.18 ± 21.23	48–103	
BMI	21.67 ± 4.70	16.58–32.60	
Work experience (Year)	9.48 ± 12.14	2–32	
Teaching hours- Before COVID (per year)	135.17 ± 35.89	40–320	
Teaching hours- During COVID (per year)	128.97 ± 32.38	40–300	
Gender (n (%))	Male	Female	
	205 (%66.13)	105 (%33.87)	
Employment Status (n (%))	Adjunct professor	Faculty member	
	80 (%25.8)	230 (%74.2)	
Education level (n (%))	Specialist	PhD	MSc
	87 (%28.07)	145 (%46.77)	78 (%25.16)

Table 3 summarizes the descriptive and analytical findings about the amount of time spent utilizing educational devices and duration of physical activity in DE. Before and during the COVID period, the mean ± SD of daily minutes of physical activity were 33.62 ± 28.46 and 24.89 ± 21.55, respectively. Between the minutes of physical activity before and during the COVID, there was a statistically significant difference (*p* = 0.022). There was an overall rise seen before and after COVID in terms of the hours spent using electronic devices in DE, which included computers, laptops, tablets, and smart phones. The amount of time spent on computers and laptops before and during COVID-19 was found to differ statistically significantly (*p* < 0.001). Nonetheless, there was not a significant difference in the amount of time spent using tablets and smartphones before and during COVID-19 (*p* > 0.05).

**Table 3:** Descriptive and analytical results of minutes of physical activity and hours of using equipment used in DE

**Variable**		**Mean**	**Std. Deviation**	**Minimum**	**Maximum**	***P*** ** Value**
Physical activity (min)	Before COVID	28.46	33.62	0.00	90.00	0.022
	During COVID	21.55	24.89	0.00	90.00	
Smartphone use (hour)	Before COVID	3.14	6.88	1.00	10.00	0.092
	During COVID	4.31	7.11	1.00	12.00	
Tablet use (hour)	Before COVID	2.57	3.38	1.00	6.00	0.763
	During COVID	2.82	3.45	1.00	8.00	
Computer use (hour)	Before COVID	4.18	6.92	2.00	12.00	< 0.001
	During COVID	6.85	8.42	1.00	14.00	
Laptop use (hour)	Before COVID	3.39	3.91	1.00	15.00	< 0.001
	During COVID	6.11	7.16	1.00	17.00	

**Fig. 1:**
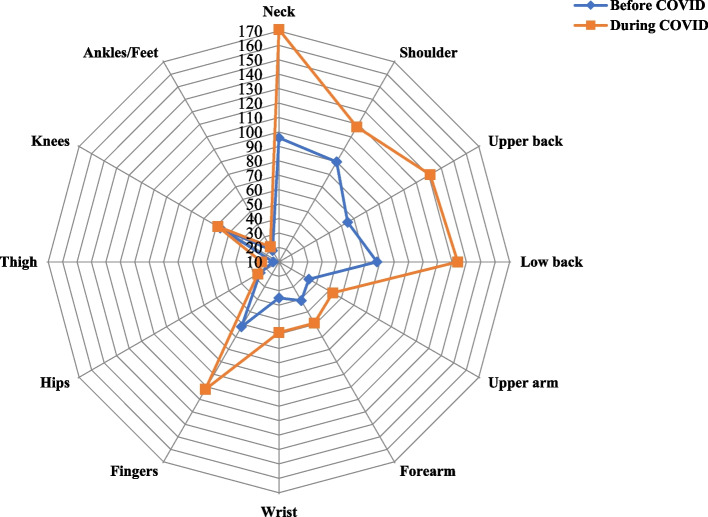
Descriptive results of the prevalence of MSDs before and during COVID

Figure 1 depicts the descriptive findings about the prevalence of MSDs both before and during the COVID period. Table 4 provides details on the cumulative incidence rate as well as comparisons of the prevalence of MSDs before and during COVID-19. Before COVID-19, the neck (30.96%), shoulders (29.03%), and low back (25.16%) were the regions with the highest rates of MSDs. During COVID, the neck (55.16%), upper back (42.25%), low back (43.22%), and shoulders (38.06%) were shown to have the highest prevalence of MSDs. The neck (Cin = 24.20%), upper back (Cin = 21.29%), low back (Cin = 18.06%), and fingers (Cin = 16.13%) had the highest incidence of MSDs.

**Table 4:** Cumulative incidence rate and comparative results of prevalence of MSDs before and during COVID

	**Prevalence**		**Incidence**		**Statistics**	
Variables	**Before COVID** ***n*** **(%)**	**During COVID** ***n*** **(%)**	**New cases**	**C**_in_^a^	**χ** ^**2**^	***P *Value** * *
**Neck**	96 (30.96)	171 (55.16)	75.00	24.20%	26.53	*P* < 0.001
**Shoulder**	90 (29.03)	118 (38.06)	28.00	9.03%	15.19	*P* < 0.001
**Upper back**	65 (20.96)	131 (42.25)	66.00	21.29%	23.16	*P* < 0.001
**Low back**	78 (25.16)	134 (43.22)	56.00	18.06%	21.84	*P* < 0.001
**Upper arm**	34 (10.96)	53 (17.09)	19.00	6.13%	8.98	*P* < 0.009
**Forearm**	41 (13.22)	59 (19.02)	18.00	5.80%	8.64	*P* < 0.011
**Wrist**	35 (11.29)	59 (19.03)	24.00	7.74%	14.32	*P* < 0.001
**Fingers**	62 (20.00)	112 (36.13)	50.00	16.13%	19.47	*P* < 0.001
**Hips**	25 (8.00)	27 (8.71)	2.00	0.64%	0.813	0.795
**Thigh**	14 (4.50)	22 (7.10)	8.00	2.58%	1.16	0.416
**Knees**	57 (18.38)	59 (19.03)	2.00	0.64%	0.798	0.812
**Ankles/Feet**	19 (6.13)	22 (7.10)	3.00	0.96%	0.844	0.715

^a^C_in Cumulative Incidence_

^**^McNemar Test

The comparative findings showed a statistically significant difference in the prevalence of MSDs in the following areas: arms (*p* = 0.007), forearm (*p* = 0.021), wrist (*p* < 0.001), fingers (*p* < 0.001), neck (*p* < 0.001), shoulders (*p* < 0.001), upper and lower back (*p* < 0.001), and fingers (*p* < 0.001). However, there was no statistically significant variation in the prevalence of MSDs in the knees (*p* = 0.812), ankles/feet (*p* = 0.715), hips (0.795), and thighs (*p* = 416) between the before and during COVID-19 periods.

Table 5 presents the findings of the variables influencing the incidence of MSDs in medical professors during the COVID-19 pandemic. A statistically significant correlation was found between the prevalence of MSDs and the increase in computer and laptop usage among the electronic devices used for DE during the COVID-19 pandemic (*P* < 0.001). There was no statistically significant correlation found between education level and the prevalence of MSDs (*P* = 0.134), however the frequency was higher in PhDs (26.1%) than in Specialists (14.83%) and MScs (13.22%). The frequency of MSDs was shown to be statistically correlated with age (*P* = 0.027), work experience (*P* = 0.016), and employment status (*P* = 0.042).

**Table 5:** Factors associated with incidence of MSDs during the COVID pandemic in medical professors

**Variable**		**MSDs**		**Non-MSDs**		***P*** Value*
		***n***	**%**	***n***	**%**	
Most frequently used types of display	smartphone	170	54.83	140	45.16	0.071
	Tablet	33	10.64	21	6.77	0.631
	Laptop	206	66.45	90	29.03	*P* < 0.001
	Computer	241	77.74	69	22.25	*P* < 0.001
Education level	Specialist	46	14.83	41	13.22	0.134
	PhD	81	26.12	64	20.64	
	MSc	41	13.22	37	11.93	
Employment Status	Faculty member	179	57.74	51	16.45	0.042
	Adjunct professor	34	10.96	46	14.83	
Work experience	Less than 10 year	38	12.25	122	39.35	0.016
	10–20 year	69	22.25	36	11.61	
	More than 20 year	37	11.93	8	2.58	
Age	Less than 40 year	48	15.48	102	32.90	0.027
	40–50 year	73	23.54	57	18.38	
	More than 50	24	7.74	6	1.93	
Computer/Laptop distance	Less than 32 inch	110	35.48	82	26.45	0.814
	More than 32 inch	61	19.67	57	18.38	
Smartphone/Tablet distance	Less than 12 inch	105	33.87	60	19.35	0.047
	12–14 inch	67	21.61	31	10.00	
	More than 14 inch	11	3.54	34	10.96	
Lighting	Good	72	23.22	48	15.48	0.271
	Medium	101	32.58	69	22.25	
	Low	12	3.87	6	1.93	
Workstation (Smartphone-Tablet)	Sitting on the ground (The Spine sloping forward)	73	23.54	40	12.90	0.031
	Sitting on the ground (The Spine is straight)	7	2.25	6	1.93	
	Sitting on the chair (The Spine slopping forward)	8	2.58	6	1.93	
	Sitting position on the chair (The spine is straight)	11	3.54	11	3.54	
	Supine position (Lying down)	70	22.58	40	12.90	
	Prone position (Lying on the stomach)	25	8.06	12	3.87	
Workstation (Computer)	ergonomic	42	13.54	66	21.29	*P* < 0.001
	Non-ergonomic	139	44.83	58	18.70	
Workstation (Laptop)	Laptop on the table (ergonomic condition)	15	4.83	41	13.22	*P* < 0.010
	Laptop on the table (Non ergonomic condition)	70	22.58	25	8.06	
	Lying on the ground	30	9.67	11	3.54	
	Laptop on the small portable tables	35	11.29	45	14.51	
	Using a laptop on the sofa	19	6.13	14	4.51	
	Sitting on the ground (Laptop on the knee)	25	8.06	9	2.90	
Head position (Smartphone-Tablet)	Neck is straight	11	3.54	16	5.16	0.029
	Neck bending up to 15 degrees	60	19.35	33	10.64	
	Neck bending up to 30 degrees	52	16.77	42	13.54	
	Neck bending up to 45 degrees	56	18.06	16	5.16	
	Neck bending up to 60 degrees	14	4.51	9	2.90	
Display height (Computer/Laptop)	Standard (Neck is straight)	76	24.51	41	13.22	0.045
	Low (neck flexion)	106	34.19	51	16.45	
	High (neck extension)	11	3.54	8	2.58	
Smartphone holding and typing method	Two-handed holding- typing with the index finger	58	18.71	35	11.29	0.628
	Two-handed holding—typing with two thumbs	24	7.74	16	5.16	
	One-handed holding- typing with the thumb finger	103	33.22	82	26.45	
Physical activity	Less than 15 min	117	37.74	50	16.13	0.006
	More than 15 min	76	24.51	66	21.29	

*﻿chi-square test (χ^2^)

The computer/laptop distance and MSDs did not significantly correlate (*P* = 0.814). A statistically significant correlation was found between the distance of smartphones and tablets from the body and MSDs, indicating that the prevalence of MSDs increased as the distance decreased (*P* = 0.047). There was no statistically significant correlation found between the prevalence of MSDs and lighting (*P* = 0.271) or between the typing method and smartphone holding (*P* = 0.628).

The prevalence of MSDs was found to be statistically correlated with the following: workstation (smartphone-tablet) (*p* = 0.031), workstation (computer) (*P* < 0.001), workstation (laptop) (*P* < 0.001), head position (smartphone-tablet) (*P* = 0.029), display height (computer/laptop) (*P* = 0.045) and physical activity (*P* = 0.006).

It should be mentioned that the majority of participants (89.6%) thought that the university's workstation had better ergonomics than their own at home.

## Discussion

During the COVID-19 pandemic, quarantine proved to be a successful strategy for preventing the virus's transmission. Nevertheless, university professors encountered difficulties as a result of the switch from in-person to DE. The purpose of this research was to determine the frequency of MSDs among university professors during the COVID-19 pandemic, compare it to the time prior to the pandemic, and find any new instances of these disorders caused by DE. Furthermore, an attempt was made to determine a relationship between MSDs and the risk factors that contribute to these disorders. Quarantine and the switch from conventional teaching to DE teaching methods have impacted the prevalence of MSDs negatively, according to the study's overall findings, which has raised cumulative incidence during the COVID-19 period. The rise in MSDs among university professors has been attributed to a number of factors, including the initiation of changes to workstation ergonomics, increased use of electronic devices compared to pre-pandemic times, and the conversion of dynamic tasks to static during online teaching or offline content creation. Put otherwise, the study's two hypotheses were supported.

The study's findings show that during the COVID-19 pandemic, there was a notable rise in the number of hours that people used computers, laptops, and smartphones. This rise can be linked to the change in teaching methods used by university professors in the pandemic versus before the pandemic. In order to prepare and present educational content online, university professors involved in DE have to spend numerous hours using desktops, laptops, and mobile devices. It would be difficult to get satisfactory DE quality without using these appliances. Therefore, the increase in the use of electronic devices during the COVID-19 pandemic is regarded as an inevitable consequence [13, 14]. These findings align with other studies conducted during the COVID-19 pandemic, where an increase in the duration of electronic device usage has been reported across various studies [40–44].

Among the most significant findings of the current study are the high prevalence of MSDs in the knees, neck, and shoulders before to the COVID-19 pandemic and the increased prevalence of MSDs in the neck, shoulders, and lower back during the pandemic. A statistically significant rise in the prevalence of MSDs has also been observed in a number of body areas, including the neck, shoulders, arms, forearms, wrists and fingers, buttocks, and thighs. Put another way, 75% of the body areas under study had a significantly higher prevalence of MSDs a year after the quarantine. The neck, shoulders, and upper and lower back regions were also linked to the highest cumulative incidence rateIn summary, university professors' necks, shoulders, and lower backs were primarily impacted, both before and during the COVID-19 pandemic. It is often acknowledged that workers in office environments and those who must use digital devices continuously for work are vulnerable to MSDs [45]. Office workers and university professors perform many of the same tasks in their careers. University professors in the medical sciences have a variety of duties, including administrative duties as well as clinical work in the healthcare system and preventive field work. Inherently, administrative work involves static body posture and extended sitting, which has been linked to upper body pain, specifically in the neck, shoulders, and lower back, according to recent studies [46].

A poorly designed workstation and long hours of sitting can cause prolonged static muscular contraction, which can alter the curvature of the spinal column and cause pressure on the intervertebral discs, muscle tension in the ligaments and tendons, and decreased tissue flexibility. Ultimately, these changes may contribute to an increased risk of MSDs [47]. These may be the reasons for the high prevalence of MSDs in the neck, shoulders, upper and lower back areas prior to and during the COVID-19 pandemic. Long-term clinical activity preceding the COVID-19 pandemic may potentially be responsible for the greater prevalence of MSDs in the knees before the pandemic [36]. Furthermore, a higher prevalence of musculoskeletal discomfort in the knee region is to be expected because university professors work in a combination of sitting and standing, in contrast to office workers who mostly sit [48]. The reduction in the prevalence of knee pain during the COVID-19 pandemic, due to changes in seated-standing work postures, supports this notion. These results agree with those of other investigations. According to Jafari et al. [35], there was a significant increase in the prevalence of MSDs during the COVID-19 pandemic compared to previous times, with the lower back, neck, knees, and shoulders having the highest prevalence among university professors. The neck, upper back, and lower back regions were shown to have the highest prevalence of MSDs during the COVID-19 pandemic among computer users working from home, according to a study conducted in India by Kulshrestha et al. [49]. The prevalence of MSDs in university professors before and during the COVID-19 epidemic was investigated as well by Rambod et al. [36], who reported a significant rise in prevalence in most body regions, including the lower back, neck, shoulders, wrists, and knees. However, no research has been conducted to determine the cumulative incidence of MSDs in university professors or to determine the number of new cases, which makes it challenging to compare these findings with those of other studies.

As previously noted, during a year of the COVID-19 pandemic-related quarantine, the prevalence of MSDs had significantly increased in 75% of the areas under study. Occupational, demographic, and biomechanical factors were measured, and statistical tests were used to determine their correlation with the prevalence of MSDs in order to determine the reasons behind the high cumulative incidence and increased prevalence of these disorders in the neck, shoulders, upper back, and lower back areas. The section's findings showed a significant correlation between the rise in MSD prevalence and factors such as prolonged computer and laptop use, employment status, age, and work experience, as well as factors like reduced distances between smartphones and tablets and faces of individuals, posture when using smartphones and tablets, posture when working with computers and laptops, head posture when using smartphones and tablets, screen height of laptops and computers from desk surfaces, and decreased physical activity. As evident, the COVID-19 pandemic and the shift in workplace from university offices to homes have led to noticeable changes in effective working conditions for the musculoskeletal system. In particular, the professors this study investigated thought that workstation ergonomics at universities were better to those at homes. These changes include longer periods spent using computers, laptops, and smartphones as a result of switching from in-person to virtual teaching methods; working in less suited environments than offices; incorrect head and neck positioning during offline content creation or virtual teaching; and improper screen height of computers and laptops from desk surfaces, all of which can result in uncomfortable postures when using smartphones, computers, and laptops [50, 51]. In comparison to before the pandemic, the decrease in physical activity during the COVID-19 pandemic has also had a detrimental impact on the recovery of muscles [52, 53]. Overall, the main cause of the increased prevalence of localized and cumulative incidences in various areas of the body appears to be the apparent shift in professors' teaching duties from dynamic to static tasks prior to and during the COVID-19 pandemic, in addition to adverse environmental factors. According to a study by Junaid Amin et al. on university professors in Saudi Arabia [54], the COVID-19 pandemic significantly contributed to the increased incidence of MSDs in this population. This is mainly because of working in poor and prolonged physical conditions as a result of lifestyle changes and DE, which resulted in less physical activity during the COVID-19 pandemic. In another study, Ghasemi et al. [40], found that a rise in MSDs among university professors can be attributed to a decrease in physical activity, an increase in sedentary work conditions, and an increase in the use of electronic devices for virtual teaching. The results of the current study are consistent with those found by Abdul Rahim et al. [55], that also identified risk factors for MSDs in teachers and university professors. These risk factors included increased age, teaching experience, long working hours, working in unfavorable postures, stress, anxiety, and fear.

Certain limitations of the current study should be taken into account. It was conducted in a cross-sectional manner, comparing the results of two periods: before the COVID-19 pandemic and over a year after the onset of quarantine due to the pandemic. It should be mentioned that it could be problematic to determine cause-and-effect interactions in cross-sectional studies. Also, the study's data were self-reported and gathered online, which means that participant errors could have affected the findings. Self-reported questionnaires may be biased or contain inaccurate data, and respondents may exaggerate or underestimate their symptoms of MSDs. Nevertheless, by including visual aids, we tried to reduce the impact of question misinterpretation in this study. The results of this study suggest that DE has led to an increase in the prevalence of MSDs. However, this conclusion should be approached with caution. It's important to consider that, in addition to DE, other factors could act as confounding variables affecting the increase in MSDs, which were not investigated in this study. For example, the social and psychological effects resulting from the COVID-19 pandemic may also influence MSDs. When generalizing the findings to other populations, care should be used because this study concentrated on a particular group of Iranian university professors. In this study, besides examining the prevalence of MSDs, we also investigated factors related to it during the COVID-19 pandemic. These results can effectively highlight the ergonomic changes introduced in the workstations of university professors due to working from home and DE. The results of this study can be utilized to identify modifications made to university professors' workstations during DE and implement them into their future professional development when comparable conditions arise. Future longitudinal cohort studies and participant follow-ups can provide more realistic results, and it is recommended to be considered in future research endeavours.

Finally, University professors can create favourable ergonomic conditions for their workstations during DE by following recommendations:

- 1- Adjusting the brightness of the workstation and preventing glare on the monitor surface
- 2- Top of monitor at or just below eye level.
- 3- Head and neck balanced and in-line with torso.
- 4- Shoulders relaxed.
- 5- Elbows close to body and supported.
- 6- Lower back supported.
- 7- Wrists and hands in-line with forearms.
- 8- Adequate space for keyboard and mouse.
- 9- Micro-breaks lasting 20 seconds every 20 minutes and mini-breaks lasting three-to-five minutes, at least every hour.

Paying attention to these recommendations and setting up a workstation with a computer, laptop and smartphone can lead to a reduction in MSDs during DE in the future.

## Conclusion

This study shows that the COVID-19-related quarantine and the move from conventional in-person education to distance education have caused a notable rise in the prevalence of musculoskeletal disorders among university professors. These disorders predominantly occur in the neck, shoulders, upper back, and lower back. Prolonged use of computers, laptops, and smartphones, as well as a decrease in physical activity and changes to teaching tasks during the COVID-19 pandemic, has all been related to these disorders. According to the findings, increasing the awareness of professors regarding the biomechanical risks arising from changes in teaching methods can contribute to mitigating the negative effects of the transition to distance education on the musculoskeletal system. These findings could be applied to improve instructors' working conditions in distance education environments and diminish related disorders in the future. Moreover, more investigation using long-term cohort studies can help clarify the effects of these changes on university professors' health.

## Data Availability

The datasets used and/or analysed during the current study are available from the corresponding author on reasonable request.

## Abbreviations

DE: Distance education
MSDs: Musculoskeletal disorders
WHO: World Health Organization
Cin: Cumulative incidence
